# Development of an automated 3D high content cell screening platform for organoid phenotyping

**DOI:** 10.1016/j.slasd.2024.100182

**Published:** 2024-09-06

**Authors:** Suleyman B. Bozal, Greg Sjogren, Antonio P. Costa, Joseph S. Brown, Shannon Roberts, Dylan Baker, Paul Gabriel, Benjamin T. Ristau, Michael Samuels, William F. Flynn, Paul Robson, Elise T. Courtois

**Affiliations:** a The Jackson Laboratory for Genomic Medicine, Farmington, CT, United States; b Yale School of Medicine, Yale University, New Haven, CT, United States; c Department of Biomedical Engineering, School of Engineering and Applied Sciences, Yale University, New Haven, CT, United States; d Department of Pharmaceutical Sciences, School of Pharmacy, University of Connecticut, Storrs, CT, United States; e Department of Urology, UConn Health, Farmington, CT, United States; f Departments of Genetics & Genome Sciences and Cell Biology, UConn Health, Farmington, CT, United States; g Department of Obstetrics and Gynecology, UConn Health, Farmington, CT, United States

**Keywords:** 3D tissue culture, Organoid co-culture, High-content screening, Automation, Image-based phenotyping, High-content imaging

## Abstract

The use of organoid models in biomedical research has grown substantially since their inception. As they gain popularity among scientists seeking more complex and biologically relevant systems, there is a direct need to expand and clarify potential uses of such systems in diverse experimental contexts. Herein we outline a high-content screening (HCS) platform that allows researchers to screen drugs or other compounds against three-dimensional (3D) cell culture systems in a multi-well format (384-well). Furthermore, we compare the quality of robotic liquid handling with manual pipetting and characterize and contrast the phenotypic effects detected by confocal imaging and biochemical assays in response to drug treatment. We show that robotic liquid handling is more consistent and amendable to high throughput experimental designs when compared to manual pipetting due to improved precision and automated randomization capabilities. We also show that image-based techniques are more sensitive to detecting phenotypic changes within organoid cultures than traditional biochemical assays that evaluate cell viability, supporting their integration into organoid screening workflows. Finally, we highlight the enhanced capabilities of confocal imaging in this organoid screening platform as they relate to discerning organoid drug responses in single-well co-cultures of organoids derived from primary human biopsies and patient-derived xenograft (PDX) models. Altogether, this platform enables automated, imaging-based HCS of 3D cellular models in a non-destructive manner, opening the path to complementary analysis through integrated downstream methods.

## 1. Introduction

### 1.1. Overview

The high-content organoid screening platform described in this work was developed to expand the utility of organoid models in high throughput screening settings. Traditionally, two-dimensional (2D) tissue cultures have been used as a first-line screening tool for drug development. However, 3D tissue cultures have gained favor in recent years due to their biological resemblance to *in vivo* models [1–3]. Despite these advantages, challenges remain in widespread implementation of 3D screening protocols [4]. These challenges, specifically the automation and integration of 3D tissue culture into high-content screens, are addressed in this study. Utilizing the Hamilton Microlab^®^ VANTAGE Liquid Handling System^®^, we have automated the necessary steps for assay development of both 2D and 3D tissue cultures. In addition, we have developed an exhaustive high-content and high-throughput screening system for drug testing by combining traditional biochemical assays (via plate reader) with novel 3D image-based analysis through the Perkin Elmer Opera Phenix^™^ High-Content Screening System. The development and workflow steps are presented in the Results section.

### 1.2. Strengths and weaknesses of automated screening using organoids

Strengths and weaknesses associated with robotic and manual liquid handling procedures have been previously outlined [4] and are described in relation to the assay protocol described herein. Manual liquid handling procedures with a standard or semi-automated pipette are easy to adapt and integrate into an assay workflow. However, they are limited in that they have limited throughput, require manually repeated interaction with an operator, and are largely dependent on the experience of said operator. Robotic liquid handling procedures overcome these presented weaknesses through automation, consistent liquid transfers, and higher throughput. However, these benefits are coupled with a high barrier of entry; relative to manually-operated systems, robotic liquid handling requires a large upfront financial and personnel investment and requires a significant amount of person-time to implement and optimize workflows. In this study, we provide unique considerations for creating an automated screening protocol for organoid models.

## 2. Materials and methods

### 2.1. Organoid media

Colon tumor organoids were grown in media consisting of *h*+++ media (Advanced DMEM/F-12, 10 mM HEPES pH 7.2–7.5, 1X Gluta-MAX, 100 μg/mL Primocin, 0.1 % BSA) [5,6] supplemented with 1X B27, 1.25 mM N-Acetyl-l-cysteine, 50 ng/mL Recombinant human EGF, 100 ng/mL Recombinant human Noggin, 10 % R-Spo1 CM (R&D Systems, cat. no. 3710–001–01) [7,8], and 500 nM A83–01, and 10 mM Nicotinamide.

Bladder tumor organoids were grown in media composed of Advanced DMEM F12, FGF7 (25 ng/ml), FGF10 (100 ng/ml), FGF2 (12.5 ng/ml), NAC (1.25 mM), NIC (10 mM), A83–01 (500 nM), Y-27,632(10 μM), B27 (2 % of final volume), N2 (1 % of final volume), and Glutamax (1X) [9].

### 2.2. Colorectal tumor organoid line derivation

Patient-derived xenograft (PDX) tumor biopsies sourced within The Jackson Laboratory (JAX) JAX-PDX platform [10,11] and covered under JAX IRB Protocol 121,200,011 were used to derive colorectal cancer PDX-derived organoids (CRC-PDXO). The CO-1 and CO-2 organoid lines were derived from JAX PDX Models J000102630 [12] and TM01258, respectively. More details on the strains can be found at mice.jax.org. Briefly, PDX tissue collected in MACS storage buffer were shipped O/N at 4°C. Upon receipt, samples were immediately minced into tissue chunks <1 mm in size with a sterile scalpel. Tissue chunks were then suspended in Matrigel at 4°C and plated onto pre-warmed plates at 37°C before proceeding to the protocol outlined in General organoid culture. As a quality control measure to confirm that all established PDXO cultures consisted of human epithelial cells, qPCR and/or flow cytometry was used to quantify mouse, human and epithelial content of the cultures. These quality control details are outlined in Section 2.2.1

#### 2.2.1. Colorectal PDXO quality control

TrypLE Express was used to dissociate CRC-PDXO into single-cell suspensions for flow cytometry. For staining, 500,000 cells were resuspended in 100 μL of FACS staining buffer (BioLegend). Blocking solution (Fc block for human + mouse, BioLegend) was added and incubated for 5 min at 4 °C to prevent non-specific binding. Following this, 5 μL of each antibody (human EpCAM-PE epithelial marker, mouse H2KD-BV421, human HLA A,B,C-AF647 from BioLegend) was added as per the provider’s protocol and incubated for 30 min at 4 °C in the dark. The cells were then washed twice with PBS containing 2 % BSA and 2 mM EDTA. The stained cells were subsequently analyzed using a BD Fortessa flow cytometer. PDXO lines were also verified to be human using an adapted qPCR protocol that independently quantifies human and mouse PTGER2 [13]. Briefly, DNA was isolated from organoid cultures and amplified using PTGER2 human forward (GCTGCTTCTCATTGTCTCGG), PTGER2 mouse forward (CCTGCTGCTTATCGTGGCTG), and PTGER2 common reverse (GCCAGGAGAATGAGGTGGTC) primers. DNA isolated from human HEK293 cells and mouse lung was amplified in the same fashion to serve as controls. Standard curve samples were prepared by mixing defined percentages of human and mouse DNA. Cycle numbers obtained from PDXO culture samples were then correlated to the standard curve to obtain percent human and mouse content. The results from these quality control measures are summarized in Supplementary Figure 1.

### 2.3. Bladder tumor organoid line derivation

Bladder tumor organoids were generated from transurethral resection of bladder tumor (TURBT) samples received from UConn Health Center Department of Surgery. These were collected through the UConn Health Center Biobank, deidentified, and deemed not to meet the definition of human subjects research by the JAX IRB (IRB Reference Number: 2017–019). TURBT samples were removed from MACS buffer and washed with Advanced DMEM F12. The tissue was then minced and resuspended in Corning Matrigel and plated in 50 μL domes onto 12 well tissue culture treated plates. After domes solidified, 500 μL of media was added to each well with bladder organoid media. Minced tissue embedded in Matrigel was allowed to acclimatize to media conditions for up to 5 days or until organoid-like structures began to appear. Liberase TM (1 mg/mL) was then added to each well to dissolve Matrigel domes and minced tissue was washed and rigorously pipetted until dissociated into small groups of cells (~2–30 cells). Cell groups were then replated in 50 μL Matrigel domes and allowed to solidify in the same media.

### 2.4. General organoid culture

Plating was performed by gently mixing organoids in ice-cold Matrigel and dispensing ~24–50 μL droplets evenly across a 10 cm dish. The dish was inverted to prevent flattening of the droplets and placed into a 37°C incubator for 20 min to allow the Matrigel to solidify in a dome-like shape. After 20 min, the plates were inverted into the correct orientation and pre-warmed organoid growth media was added.

To passage organoids, media was aspirated, and ice-cold Cell Recovery Solution (CRS) was added to the plate. The domes were gently scraped off by pipetting repeatedly and the cell-CRS-Matrigel mixture was placed in a 15 mL centrifuge tube and incubated on ice for 30–45 min. After incubation, the tubes were centrifuged at 200 g for 5 min and the supernatant was removed. Organoid cultures were resuspended in *h*+++/BSA 0.1 % base media and broken apart by pipetting-mixing until most organoids had broken into multiple pieces or approximately 50 times with a 1 mL pipette tip. Single cells were removed by pulse centrifuging to 520 g for 1 second and removing supernatant. Matrigel was then added, and the organoids were passaged 1:3 or 1:4 depending on the organoid type and confluence of the culture. Organoid media was replaced every 2–3 days. Typical cultures reached confluence in 5–10 days.

Organoid cultures were also routinely tested for mycoplasma at JAX’s Molecular Diagnostic R&D Laboratory before being introduced onto the automated pipeline.

### 2.5. Organoid preparation for automated liquid dispensing

Organoids were expanded for screening experiments to achieve sufficient numbers. They were then prepared up until the point of passaging onto a new plate using the method described in General organoid culture.

Single cells were removed through pulse centrifugation as previously described in Section 2.4. Cell clumps (or dissociated organoids) were then resuspended in *h*+++ / 0.1 % BSA and gently passed through a 70 μm filter to remove large cell clumps or undissociated organoids so that a uniform size could be obtained (Supplementary Figure 2). Upon filtering, cell clumps were plated in Matrigel and allowed to grow for 3 days before preparing for automated liquid dispensing.

3-day-old organoids were used for HCS. Briefly, Matrigel was melted as outlined in the *Organoid culture* section to recover organoids. Only wide-bore tips were used at this stage to avoid disrupting the structure of the organoids. Handling of the organoids through liquid transfers was also limited as much as possible to prevent both unwanted changes in organoid morphology and generation of a large single cell population. Organoids were resuspended in organoid growth media at the desired density (~175 organoids per well), dispensed into a 24-section plate, and loaded onto a vortex plate holder on the Hamilton liquid handler before the automated protocol was started.

### 2.6. Dispensing Matrigel

Matrigel, previously stored at 4°C, was transferred to a pre-cooled 96-well reservoir using a multichannel pipette. The loaded 96-well plate was then placed into the Hamilton liquid dispenser onto a cooling block kept at 4°C to prevent polymerization of the Matrigel. A 96-well automated pipettor located in the Hamilton liquid dispenser then transferred 11 μL of Matrigel to each well of a 384-well assay plate (Perkin Elmer CellCarrier Ultra high-content screening plates) located on a thermal plate at 4°C and an automated shaking sequence began, creating a uniform bed of Matrigel at the bottom of each well. The 384-well assay plate was then heated to 37°C and the assay plate was incubated for 20 min to allow polymerization of the Matrigel. Once polymerization of the Matrigel was complete, dispensing of the organoids automatically began. At this stage, a control plate was also prepared for Day 1 data acquisition. For the purposes of defining time, Day 0 is the day at which organoids are plated onto the 384-well assay plate.

### 2.7. Dispensing organoids

Organoids were loaded onto the deck after following the steps described in Organoid preparation for automated liquid dispensing and dispensed onto a 24-section plate shaken in a vortex pattern on its deck position. The 96-well head proceeded with gentle mixing of the organoids prior to dispensing onto the polymerized Matrigel. 35 μL of organoids were dispensed into each well. The organoids were then transferred into an incubator at 37°C for 24 h to allow the organoids to sit on the bed of Matrigel and acclimate prior to initiating the drug screen.

### 2.8. Dispensing drugs

The system dilutes and randomizes up to 8 compounds: 6 drugs + 1 positive control + 1 negative control can be selected for dosing organoids per plate. To conduct the specific experiments described in this study, drugs were serially diluted over 12 concentrations on a 96-well plate, following a half-log dilutions series. They were then geographically randomized onto a 384-well plate capable of drugging up to four plate assays. Randomization of treatments across the 384-well plate minimizes edge effects and allows for optimal full-plate usage [14]. The assay plate had *n* = 4 technical replicate wells with identical drugs and concentrations of drugs. Drugs were dispensed on Day 1 of the experiment (Day 0 = organoid dispensing onto the 384-well assay plate). Drugs were dispensed into the assay plate, *i.e*. no drugs were dispensed into the control plate.

### 2.9. Dispensing fluorescent dyes

Dyes were prepared onto 96-well PCR plates at the relevant concentrations prior to loading onto the Hamilton deck. 5 μL of dye were dispensed into each well of the 384-well assay plate for a final concentration of 10 μM for calcein green AM and Hoechst and 1 μM for ethidium homodimer and TO-PRO-3. The Hamilton automatically turned off its lights and the organoids were incubated on the deck for 15 min at 37°C as part of the current programming. This protocol was performed on Day 1 for the control plate and on Day 5 for the assay plate.

### 2.10. Automated high content imaging

After dyes were added and the organoid cultures were incubated according to the manufacturer’s designated protocol, the assay plate was transferred by robotic arm (GX Robot (PAA)) to the imaging system, the Opera Phenix High-Content Screening System (Perkin Elmer), where an internal chamber keeps the plate incubating at 37°C and 5 % CO_2_. For each 384-well plate, all wells were imaged with 4 fields/well at 10X magnification. The remaining imaging settings were determined at the time of the experiment, including starting level and number of Z-stacks. These settings were optimized in preliminary experiments to determine the best dyes and imaging conditions for the organoid culture of interest.

### 2.11. Automated biochemical cell viability assay

The biochemical assay to determine cell viability was adapted from a previous study assessing colorectal organoids [15] and performed post-imaging. Briefly, Promega^®^ CellTiter-Glo 3D was used to quantitatively measure the ATP content and assess organoid viability. This reagent was prepared by pre-dispensing ~160 μL into each well of a 96-well reservoir plate. This plate was then loaded onto the Hamilton deck on the last day of the experiment, Day 5, six total days after initial plating of the organoids. The 40 μL of CellTiter-Glo was then dispensed into each well of 384-well assay plate. The plate was incubated and shaken in the dark at 37°C on the Hamilton deck. It was then transferred by robotic arm to the SpectraMax i3x to obtain luminescence readings in accordance with the manufacturer’s protocol. The luminescence readings (*n* = 3 or 4 per assay plate) were captured and exported for de-randomization. *N* = 3 was chosen for experiments in which the last replicate was saved for cell cryopreservation.

### 2.12. Cell cryopreservation

Cryopreservation was performed by cooling the assay plate to 4°C on the Hamilton deck. A precooled 96-well media plate with DMSO (10 % final concentration after addition to assay plate) and FBS was transferred to the deck and kept at 4°C on deck. After biochemical analysis was complete, the last previously undisturbed quadrant with organoids was preserved by dispensing cold cryopreservation media, pipette mixing with the Hamilton, transferring to a 96-well storage plate, and storing at −80°C.

### 2.13. Nanoparticle fabrication and assessment

Liposomal formulations were prepared using a co-axial turbulent jet in co-flow and particle size and polydispersity (PDI) were determined by dynamic light scattering (DLS) as previously described [16]. The liposomal formulation consisted of phosphatidylcholine, hydrogenated (HSPC), 1,2-distearoyl-sn-glycero-3-phosphoethanolamine-N-[amino (polyethylene glycol)−2000] (ammonium salt)1,2-distearoyl-sn-glycero-3-PE (DSPE-mPEG2000) and cholesterol in 10 mM histidine at pH 6.5. The molar lipid ratio was HSPC/Cholesterol/mPEG2000-DSPE (56.3:38.4:5.3 mol%) with a final lipid molarity of 20 mM. A loading batter of 250 mM Ammonium Sulfate was used to load doxorubicin-HCl and the concentration of doxorubicin-HCL was approximately 2 mg/mL.

### 2.14. Data analysis

#### 2.14.1. Derandomization

Randomization with a Mersenne twister algorithm was performed and tracked using Hamilton HSL. Derandomized data was sorted using Excel.

#### 2.14.2. Dose response curves

Dose response curves were generated by normalizing luminescence readings or calculated live organoid area by the average of the control-treated wells (% viability) or by normalizing the percentage of live organoids within a well by the same quantity measured in control-treated wells (% effect). All graphed curves were generated using GraphPad Prism v9.

#### 2.14.3. GR50 metrics

Growth rate (GR) metrics provide a way to assess drug response that is independent of the division rate of the cell cultures being assayed [17]. In this study, this metric is calculated by either biochemical assays or through imaging. The biochemical method has been adapted from a previous study [18] and uses a Day 1 (drug addition day) plate to assess baseline ATP before treatment and then repeat assessment of a separate assay plate at Day 5 (final day) to determine GR metrics. The GR metrics are also calculated in this study through image-based determinations of live and dead organoid area using Day 1 and Day 5 high-content imaging data.

## 3. Results

### 3.1. Workflow design

First, Matrigel is plated into an assay plate (Supplementary Figure 3, step 1) and allowed to solidify at 37°C on a heat block on the Hamilton deck. The Matrigel growth matrix notably solidifies with a meniscus in the wells of a 384-well plate (Supplementary Figure 3). After organoids have been transferred into an organoid reservoir plate, as seen in (Supplementary Figure 2, step 5), they are ready to be pipetted.

**Fig. 1.**
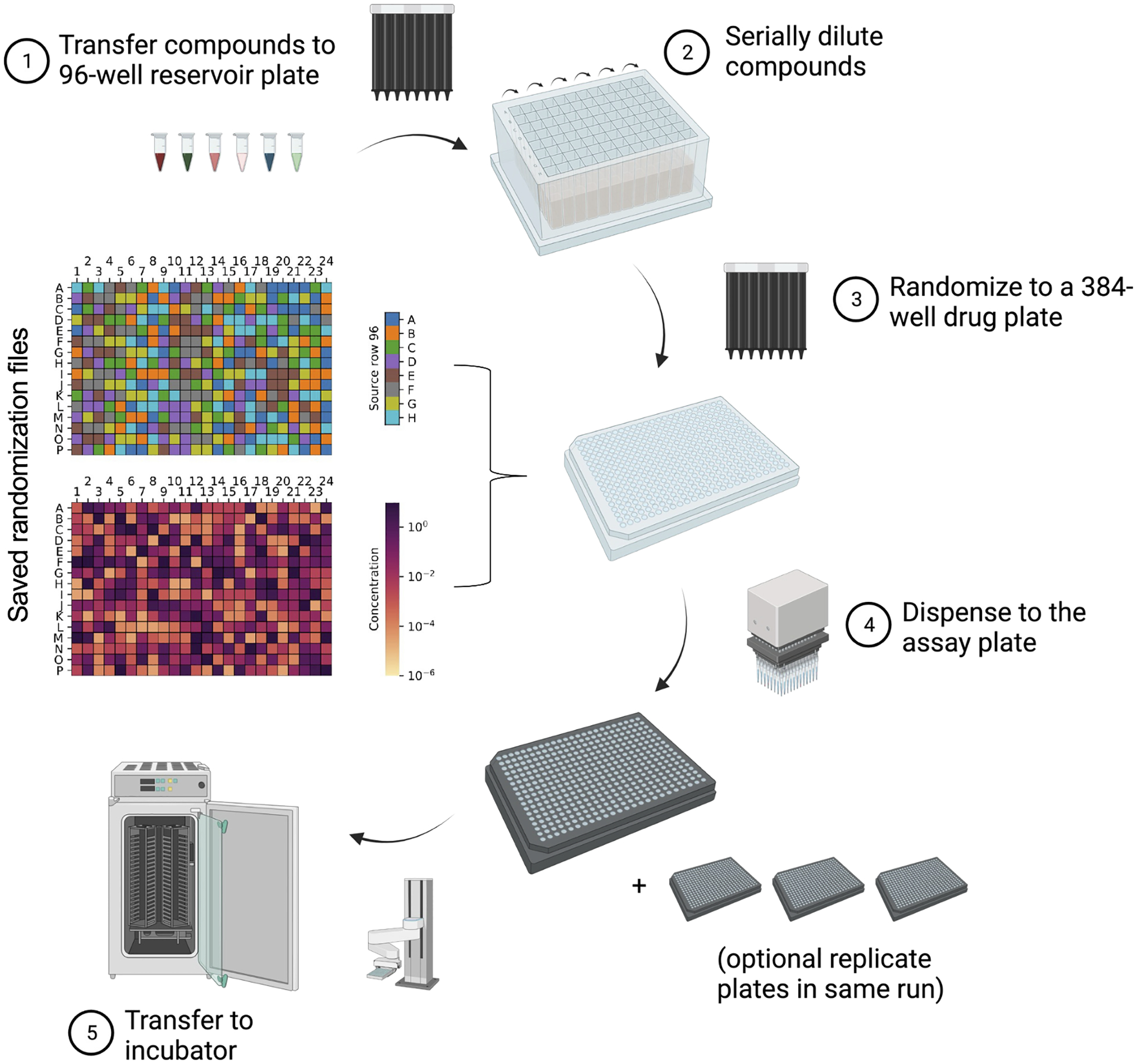
Compound transfer, dilution, and randomization is integrated between system components. **(1)** Compounds are transferred from stocks and **(2)** serially diluted before being **(3)** randomized on a 384-well compound reservoir plate. Compounds are then **(4)** pipetted into the assay plates with a 96-well head. The assay plates are then **(5)** transferred into an automation-compatible incubator.

After pipetting of organoids onto the solidified Matrigel, the assay plate may be transferred into an incubator. At the appropriate time, further addition of materials may be performed, as outlined in Fig. 1.

### 3.2. Robotic liquid handling vs manual pipetting

Unique considerations were accounted for to integrate organoids into the automated workflow. For example, Matrigel possesses unique physiochemical properties that allow it to remain liquid at 4°C but adopt a solid, gel-like consistency at 37°C [6]. Our initial tests revealed that automated dispensing of Matrigel is challenging due to premature solidification of Matrigel (not shown). To ensure even automated pipetting across a 384-well assay plate, several steps were undertaken. First, a thermal plate adapter was constructed to keep the 96-well Matrigel reservoir plate at 4°C. Second, the feasibility of manipulating the physiochemical properties of Matrigel to achieve superior dispensing was explored through dilution with PBS buffer.

**Fig. 2.**
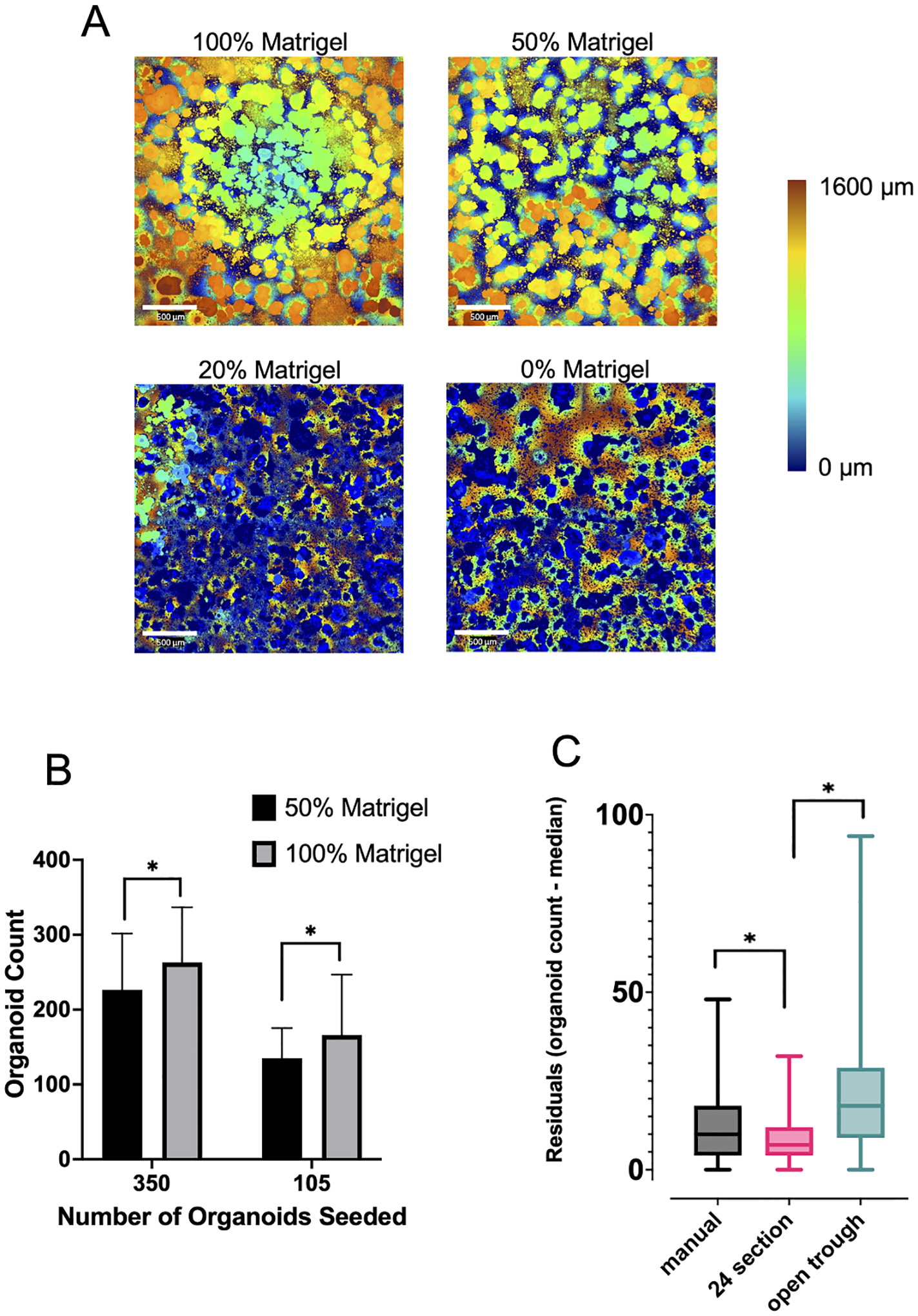
Various factors contribute to the dependability of the automated experimental workflow and must be optimized prior to beginning experiments. (A) A heatmap of organoid position along the Z axis from the bottom of the assay plate to the top of the culture shows significant differences in organoid position depending on % of the culture comprised of Matrigel. (B) Organoid growth is affected by culture conditions, including the % Matrigel that they are plated with. 100 % Matrigel is more successful than 50 % Matrigel in growing organoids, irrespective of the number of organoids initially plated. (C) Different consumables (24-section plate, open trough plate) were tested to optimize organoid dispensing and compared to manual dispensing. Residuals were calculated by taking the absolute value of (target organoid number – organoid count in the well). Target organoid number was ~200 for these experiments with an *n* = 384 wells * denote p-value < 0.05 with an *n* = 384 wells. Scale bars = 500 μm in panel A.

To test the necessity of growing organoids during a short duration (3 days) in pure Matrigel, the growth matrix was diluted with PBS buffer. After dispensing Matrigel and incubating at 37°C to ensure solidification, organoids were dispensed directly on top of the solidified Matrigel (Day 0). After three days of growth in the matrix (Day 3), analysis was completed to determine organoid level within the Matrigel. Organoids were imaged, segmented, and quantified on max-projected stitched images for each well, using the Opera Phenix Harmony software. At 0 % and 20 % Matrigel, organoids sank to the bottom of the plate after dispensing as seen in Fig. 2A. At 50 % Matrigel, organoids largely remained on top of solidified Matrigel. However, organoid count data showed that a higher number of organoids were grown from 100 % Matrigel compared to 50 % Matrigel Fig. 2B, suggesting that the growth of organoids is dependent on the concentration of Matrigel initially plated.

This step was also critical in optimizing imaging stages of the workflow. Minimizing the number of Z-stacks needed to image all the organoids in the culture was important to efficiently utilize the microscope. Imaging studies are often also complicated by the growth matrix meniscus effect shown in the single well illustration in Supplementary Figure 2. Organoids initially lay on the solidified Matrigel growth matrix. This allows shorter imaging durations due to a smaller Z-stack window in which organoids must be imaged. As the organoids grow in space, the Z-stack window expands, and imaging requires more time and generates more data. This serves as an important consideration when determining experiment length.

Additionally, the assay plates experience a well-documented technical artifact—the “edge effect”. This occurs because organoids are grown over six days in a limited media volume per well. Media evaporates from the outer edges of the plate, significantly impacting the final results [19,20]. One can partially mitigate this effect by utilizing the outer 76 wells of a 384 well plate as media controls, though this greatly reduces the usable wells per plate. An alternative mitigation strategy is to randomize the location of experimental conditions across the assay plate, though this typically requires tedious, single-channel pipetting. One major benefits of robotic liquid handling is the ease of randomizing experimental conditions, *e.g*. drugs and dosages, across the full 384-well plate. This allows for incorporation of drug randomization across the 384-well plate, a method which maximizes plate economy and minimizes the introduction of discrepancies by distributing error [14].

### 3.3. Organoid dispensing considerations

In addition to the unique properties of Matrigel, the cell-cell adhesions present within organoids makes them challenging to dispense, as they are notably “sticky” during transfers. To understand the optimal way to dispense these organoids, several source plates and procedures were characterized. Fig. 2C shows three methods that were tested and highlights that the 24-section plate was the optimal plate for the automated protocol.

Organoid size also plays a critical role in the robustness of a robotic pipetting system and its downstream use in high content screening. There are multiple approaches to standardizing the starting size of organoids, each with tradeoffs, that have been implemented across various starting cell types including adult stem cells (ASCs) as either tissue fragments or single cell suspensions [21–23], pluripotent stem cells (PSCs) [24–26], or fetal progenitor cells [27,28]. A common approach is to start from single cell suspensions where tissue fragments are enzymatically and mechanically dissociated. The benefit of this method is that it is relatively easy to perform and normalizes the starting organoid size. However, organoid growth from single cells requires a longer duration of time to reach adequate maturity, quantity, and size. It also leads to heterogenous sized organoids that may be undesirable when attempting to standardize an assay.

Although it may be possible to adequately address these challenges, we choose to start from already formed organoids as opposed to single cells. Starting from pre-formed structures allows organoids to grow in a short time frame, retains the initial cell type and state diversity, requires minimal organoid manipulation, and has been used previously to model disease in a high throughput manner [29]. However, starting from tissue fragments results in a diversity of organoid sizes which presents challenges in dispensing, expansion, and downstream analysis. Namely, organoids that start at a large size or that are grown indefinitely exhibit cell stress responses due to lack of vasculature and poor media perfusion to cells in the center of their structure [30]. In addition, observations made early on during optimization (not shown) revealed that imaging studies are complicated by wide distributions in organoid size; organoids that start at a larger size tend to dominate the culture, making it difficult to assess drug response from smaller organoids.

We addressed these challenges using sieve filtration to standardize initial cluster sizes. We also generated advanced automated shaking and pipetting protocols for the Hamilton, which allowed for programming of pipette volume, speed of aspiration, speed of dispensing, robotic movement speed, duration of plate shaking, rigor of plate shaking, and pipette level within the assay plates. Important details included speed of aspiration and dispensing, each of which directly influenced the proper mixing and precision of dispensing of organoids and are outlined in Supplementary Figure 4 for all liquid classes used. For example, colorectal organoids (cystic) dissociated more freely than bladder organoids (non-cystic), making it necessary to adjust the number of pipette mixing steps to retain organoid structural integrity and minimize breakdown. Consideration of critical parameters, including Matrigel concentration, automation plates, and starting organoid number are described in Fig. 2.

In this optimization experiment, manual dispensing was performed by securing an 8-channel trough to a vortex mixer and mixing at a low speed while dispensing organoids into a 384-well plate with a semi-automatic 8-channel pipette. The “24-section” plate and “open trough” plate were placed into the Hamilton Vantage Liquid Handler and set to shake in a figure-8 pattern at 300 rpm. Organoids were dispensed during active shaking into a 384-well plate. Significant variations in number of organoids plated were seen across the 384-well plate when the open trough plate was used, as seen in Fig. 2C.

### 3.4. Biochemical assays vs imaging assays

To assess the strengths of each method, their sensitivity in detecting changes within organoid cultures was compared using automated protocols. In general, imaging experiments require extensive optimization to acquire high-quality images and the viability assessments depend on the image analysis pipeline itself. Conversely, cell viability biochemical assays are simple to employ, require minimal optimization, but can usually only assess a single metabolite. To further understand the possible differences in a broader context, two different organoid types, CRC-PDXOs and patient-derived bladder organoids (bladder PDOs), were utilized.

**Fig. 3.**
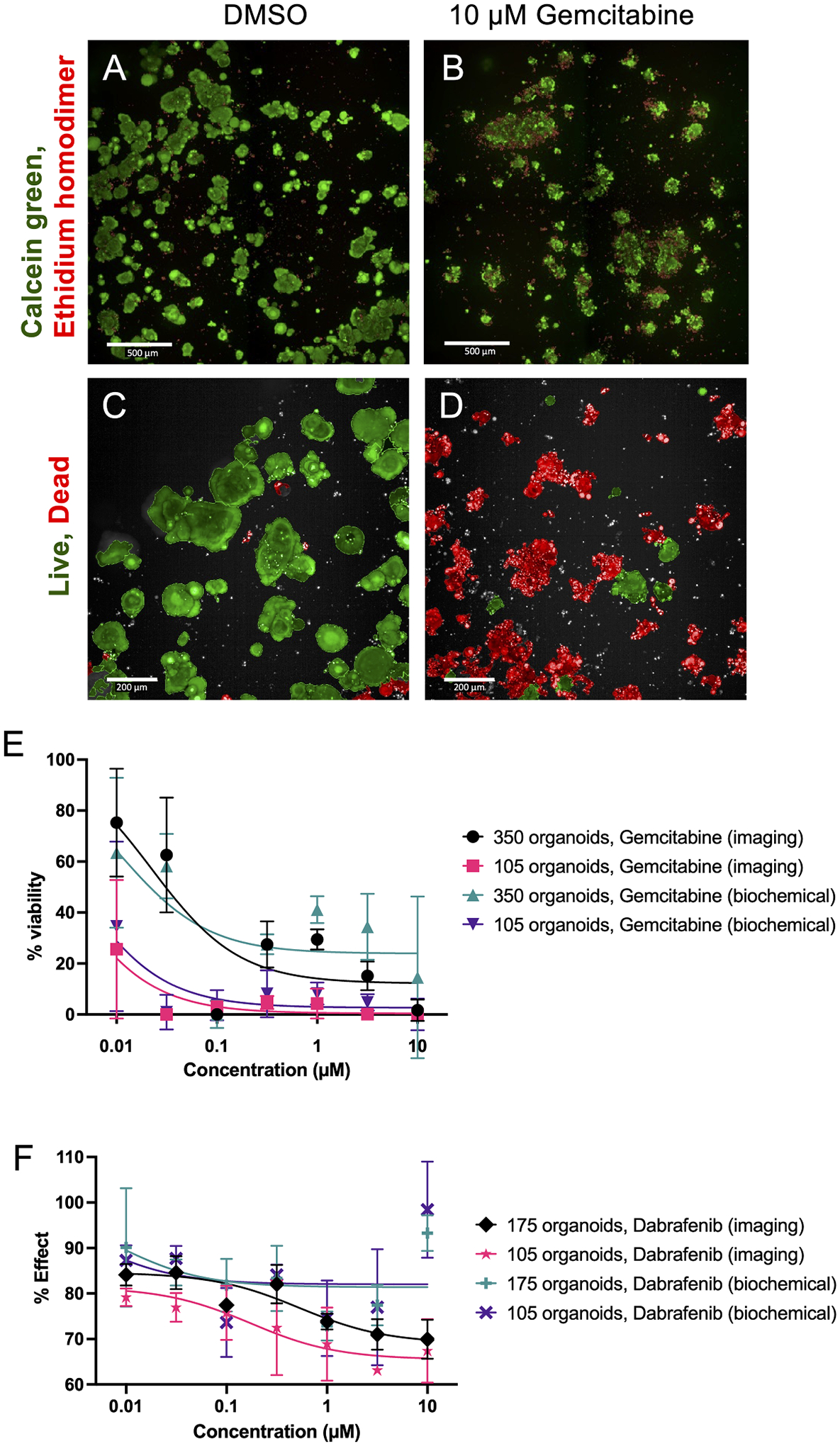
Organoid segmentation and live/dead staining are used to assess viability and drug effects using Harmony software. Calcein green is used to stain live cells while ethidium homodimer (red) is used to stain dead cells. Representative images show calcein green and ethidium homodimer (red) staining in a CRC-PDXO treated with 0.5 % DMSO (A) and 10 μM gemcitabine (B). Pseudo-colored images show live (green) and dead (red) segmented CRC-PDXO in DMSO-treated (C) and 10 μM gemcitabine-treated cultures (D). (E) depicts cell viability curves gathered from imaging and biochemical analyses of the colorectal tumor organoids when 105 organoids are originally plated or when 350 organoids are originally plated (*n* = 4 technical replicates for each condition). (F) highlights the differences between biochemical-based and imaging-based analyses when the organoids are treated with dabrafenib (*n* = 4 technical replicates for each condition). (A) and (B) scale bars = 500 μm. (C) and (D) scale bars = 200 μm.

CRC-PDXO organoids were prepared as described in the methods section. Different initial organoid densities were evaluated to determine possible variations in detectable phenotypic and biochemical changes. Organoids plated on Day 0 were treated with drug on Day 1 as previously described. On Day 6, viability dyes were added and the organoids were imaged in 3D (Fig. 3A). They were then segmented and classified as dead or alive using Harmony software as demonstrated in Fig. 3C and 3D. The organoids were then evaluated using CellTiter-Glo. In cell viability assays that assess response to gemcitabine against either 350 or 105 organoids initially plated, similar responses are seen between biochemical and imaging analysis (Fig. 3E). However, when response to dabrafenib is tested against organoids plated at different densities, imaging detects phenotypic effects at lower drug concentrations, as seen in Fig. 3F where the % effect depicted is the proportion of live organoids for a given condition.

An additional strength of automated image-based workflows is their ability to closely assess multiple factors such as organoid structure, viability, and compound internalization across a range of time points without user intervention. Supplementary Figure 5 shows the difference in drug internalization of doxorubicin-HCl and liposomal doxorubicin (Doxil, an FDA-approved drug product), through doxorubicin-HCl autofluorescence and liposomal nanoparticle delivery of doxorubicin-HCl in bladder cancer organoids (BO).

**Fig. 4.**
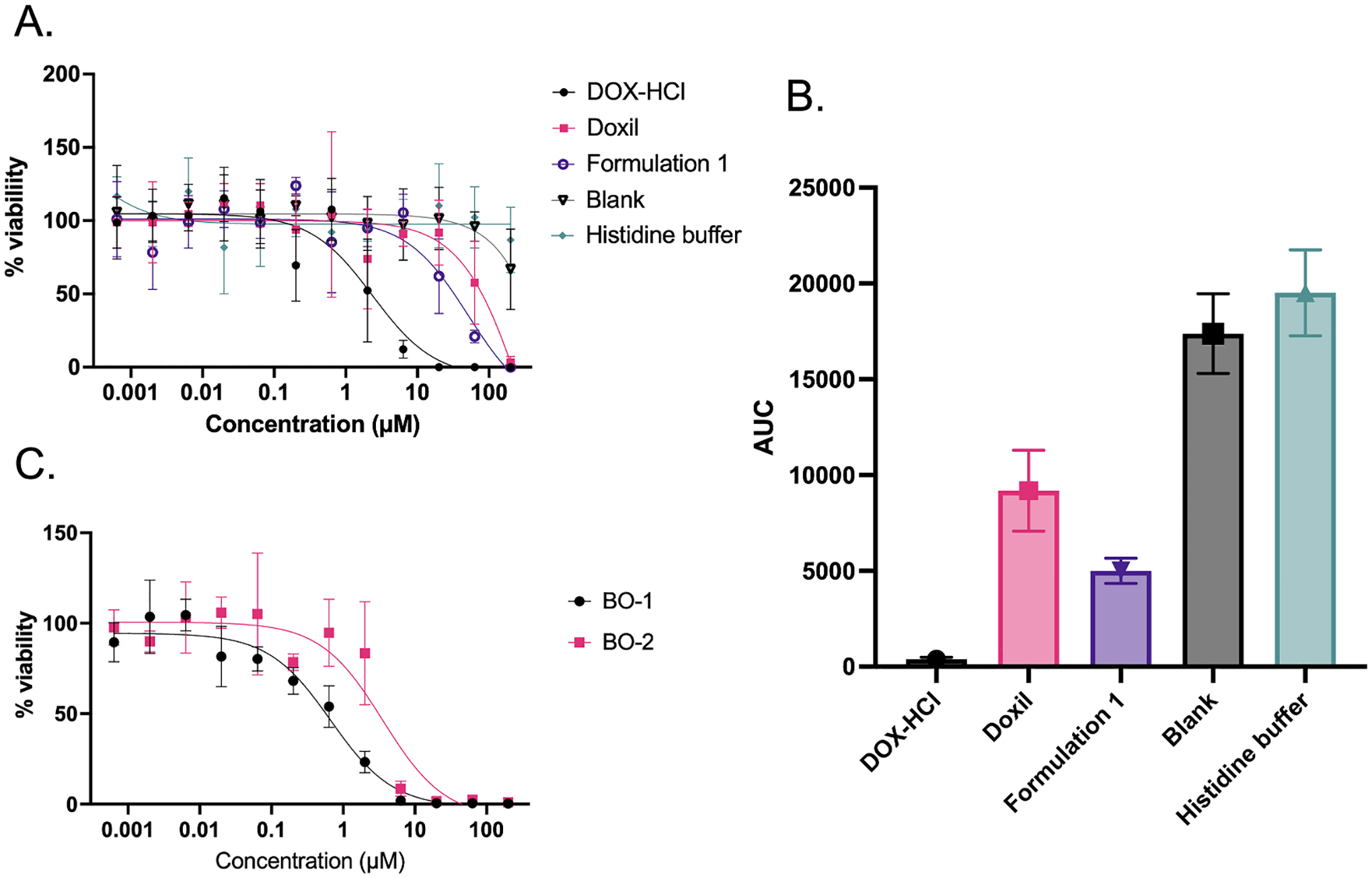
Viability curves with biochemical or imaging assays reveal differences in response to doxorubicin between bladder organoid cultures derived from two different patients (BO-1 & BO-2) and to different treatments. (A) shows BO-2′s differential responses to doxorubicin (DOC–HCl), Doxil, a proprietary liposome formulation of doxorubicin (Formulation 1), blank liposomes (Blank), and a histidine buffer control using imaging analysis (*n* = 4 technical replicates for each condition). (B) shows an area under the curve analyses for panel (A), highlighting significant differences between all treatments except between blank liposomes and histidine buffer. (C) shows difference in response to doxorubicin between two different bladder organoid lines, BO-1 and BO-2 using biochemical analysis (*n* = 4 technical replicates for each condition).

Various dyes may also be easily incorporated into the assay protocol. DRAQ5 allows visualization of the nuclei within the epithelial cells that comprise the organoids and can serve as an indication of structure and morphology. Differences in organoid response are dependent on the specific treatment given and were visualized at either 4X or 10X (Supplementary Figure 6). As expected, organoids treated directly with Doxorubicin HCl had fewer viable cells than those treated with Doxil (a polyethylene glycol-coated liposome-encapsulated form of doxorubicin), as represented by the reduction in cells that are stained with calcein violet (Supplementary Figure 6). These differences can be seen in image-based analyses of cell viability, shown in Fig. 4A. Importantly, the difference between the response to Doxil and the response to Formulation 1 can be attributed to particle characteristics and morphology as described in Supplementary Figure 7. Mainly, the morphology of doxorubicin within Doxil is an elongated nanorod crystal, giving the particles an ovular shape [31]. In contrast, the length of these nanorod crystals within Formulation 1 is shorter, giving the particles a spherical morphology (data not shown). Analyses of the significance of each response can be performed using area under the curve, or AUC analyses (Fig. 4B).

To demonstrate the robustness of the automated organoid screening system, experiments with different organoid types and different treatments were conducted. Bladder cancer organoids derived from two separate patients were tested to evaluate the adaptability of the automated workflow. Organoids were plated on Day 0, treated with drug on Day 1, and imaged/evaluated with CellTiter-Glo on Day 6.

Fig. 4C reveals the difference in response between these two bladder cancer organoid cultures, Bladder Organoid 1 (BO-1) and Bladder Organoid 2 (BO-2) from two distinct patients. Variation in these two bladder organoid lines can be attributed to biological variation related to pathological features of the tissue from which the organoid lines were derived. More specifically, BO-1 is from an invasive urothelial carcinoma with squamous differentiation and muscle invasion while BO-2 is from a high-grade urothelial carcinoma with no identifiable invasion. Since BO-1 may be a more rapidly dividing organoid line, it is reasonable to expect greater sensitivity to a DNA intercalating agent such as doxorubicin, especially treatment naïve tumors such as those used here. Imaging analysis reveals the difference in response to various treatments as shown in Fig. 4A and in the AUC analysis in Fig. 4B.

### 3.5. Co-culture experiments

To further demonstrate the versatility of this integrated automated high-content screening system, we conducted drug screens on colorectal organoid co-cultures. These cultures were plated into 384-well assay plates on Day 0, drugged on Day 1, and evaluated on Day 6 using high-content imaging and CellTiter-Glo. In these experiments, imaging assays allowed organoid segmentation and dose response analysis with growth rate (GR) metrics in place of traditional dose response (IC50) metrics for each one of the organoid lines present in the co-culture. GR metrics were made possible by preparing a separate plate and evaluating *n* = 12 wells using imaging and CellTiter-Glo analysis for normal growth rate at Day 1.

The benefit of using GR lies in its ability to overcome confounders due to baseline differences in a cell line’s rate of division [32]. The presented organoid screening system is ideal for these calculations as it can be programmed to automatically obtain viable organoid size at the beginning and end of an experiment through imaging. Supplementary Figure 8 illustrates culture of PDX-derived colorectal cancer organoid lines (CRC-PDXO). We used an independent CRC-PDXO engineered to stably express mCherry (+) (CO-1) organoids, and mCherry (−) (CO-2) organoids cultures, and a co-culture of each at the beginning of an experiment on Day 1 directly before drug treatment (CO-1+CO-2).

**Fig. 5.**
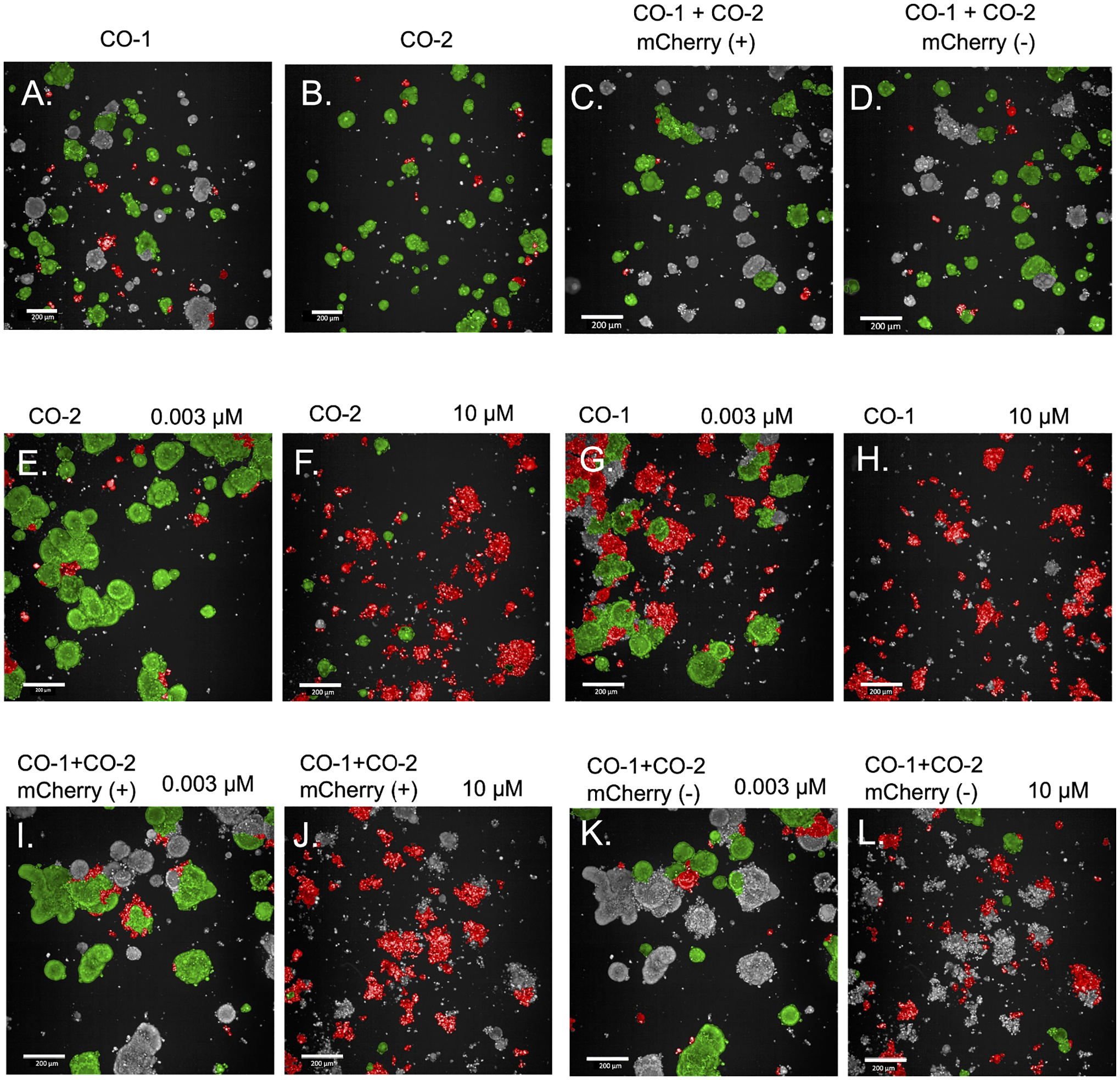
Organoids in single-cultures or co-cultures are classified into mCherry positive or mCherry negative and then classified into live (green) or dead (red). (A) Organoids that are highlighted either green or red have been classified as expressing mCherry. Green-highlighted organoids have also been classified as living while red-highlighted organoids have been classified as dead. (B) Organoids that are highlighted either green or red in this panel have been classified as mCherry (−) organoids. The green-highlighted ones are therefore mCherry (−) live organoids while the red-highlighted ones are mCherry (−) dead organoids. (C) and (D) depict a co-culture of mCherry (+) and mCherry (−) organoids plated in equal proportions. Organoids highlighted green or red in (C) show mCherry (+) organoids that are either classified live or dead, respectively. Organoids highlighted green or red in (D) show mCherry (−) organoids that are either classified live or dead, respectively. (E) and (F) show CO-2 organoids treated with 0.003 μM gemcitabine and 10 μM gemcitabine, respectively. (G) and (H) show CO-1 organoids treated with 0.003 μM gemcitabine and 10 μM gemcitabine, respectively. (I) and (J) show mCherry (+) organoids in a CO-1+CO-2 co-culture treated with 0.003 μM gemcitabine and 10 μM gemcitabine, respectively. (K) and (L) show mCherry (−) organoids in a CO-1+CO-2 co-culture treated with 0.003 μM gemcitabine and 10 μM gemcitabine, respectively. Green in all panels indicates organoids that were classified as living whereas red indicates organoids that were classified as dead. Images shown in **A-D** were gathered on Day 1 of the assay, ~24 hr after initial plating of the organoids. Images shown in **E-L** were gathered on Day 6 of the assay. Scale bar = 200 μm.

Each organoid line is segmented within the co-culture based upon its fluorescent or non-fluorescent phenotype through imaging and automated assessment of fluorescence intensity as shown in Fig. 5. Each organoid within each line is then separated based upon its initial viability directly after plating through a linear classifier in the Harmony software. While we were able to assign most of the organoids to their original line based solely on mCherry fluorescence, panel A in Fig. 5 shows some organoids remain unlabeled (gray), representative of the challenges faced in classifying organoids based upon fluorescence intensity. This challenge arises from variable expression of mCherry within and amongst organoids.

**Fig. 6.**
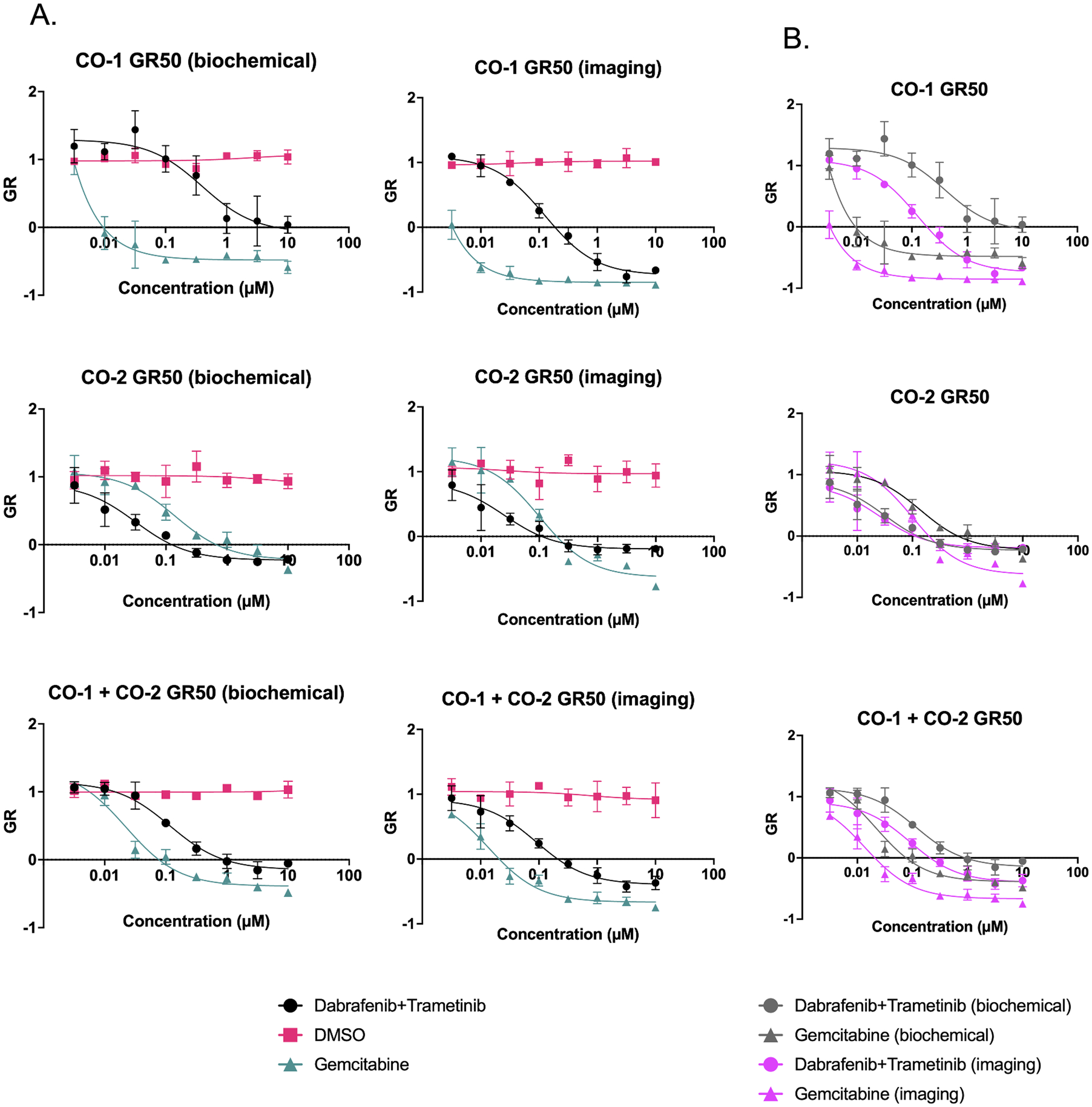
GR curves are generated using imaging and biochemical readouts after treatment with gemcitabine, combination dabrafenib/trametinib or DMSO control. Panel (A) compares GR curves generated by either biochemical or imaging modalities. (B) depicts GR curves from the same experiment overlayed on the same graph for easier comparison. *n* = 3 technical replicates for each condition depicted.

This challenge must be taken into account and is further magnified at the endpoint of the assay when cell death results in autofluorescence of mCherry negative organoids. This problem can be overcome by using different cell reporters or utilizing fluorescent cell trackers, albeit over shorter experimental durations to ensure ample expression of transient markers. Despite these obstacles, imaging analysis pipelines can be optimized using a combination of empirical methods and machine learning algorithms present in the Harmony software to generate imaging-based GR curves which closely resemble their biochemical-based counterparts, as shown in Fig. 6.

Panels A-D in Fig. 5 show the viability assessment before organoids have been treated with gemcitabine and before the target assay endpoint is reached, while Panels E-L in Fig. 5 depict after treatment and at the end of the assay. GR analysis can be run using this imaging data in combination with the data obtained at the beginning of the assay. Biochemical analysis is run on the same plate in the same culture wells directly after imaging studies have been conducted.

Direct comparison of biochemical and imaging modalities for assessing organoid viability are shown in Fig. 6A. Similar trends and response to dabrafenib + trametinib, and gemcitabine were apparent with no appreciable response in the DMSO control. The main difference seen in these assays, as was seen in our non-co-culture assays described earlier, is the sensitivity to phenotype changes in imaging studies. As seen in Fig. 6B, organoids that are analyzed through imaging reach their GR50 (intersection with the x-axis) value before organoids that are analyzed through biochemical assays.

As depicted in Fig. 6B, in the CO-2 organoid line, imaging-based response to gemcitabine and dabrafenib + gemcitabine is generally more consistent with biochemical-based response when compared to CO-1. This difference in response suggests that phenotypic changes are not entirely consistent with biochemical changes and that further investigation into these cellular processes may be warranted.

## 4. Conclusion

Robotic liquid handling is more consistent and amendable to high throughput screening than manual pipetting. Although this is an expected finding, producing a functional workflow that can be adapted to different cultures and experimental conditions while simultaneously maintaining efficiency is challenging. Programming the correct liquid handling parameters requires thorough understanding of variations in biological models and how they will likely respond to manipulation in automated workflows. Despite these challenges, the system described in this work is capable of dispensing Matrigel, viability dyes, and biochemical reagents, all while maintaining high quality assay conditions and randomizing conditions avoiding edge effects and technical biases.

A core strength of the high-content screening system described herein is its ability to be easily adapted to accommodate 3D cellular systems such as organoid lines growing as cystic (CRC-PDXO) or solid (Bladder PDO) organoids. The rigor of pipetting, speed of shaking, time for dispensing, and temperature on the instrument’s deck can be easily changed to optimize for different tissue types ensuring integrity of 3D organoids for perturbation assays. This ensures that organoids can maintain their viability to the end of an assay when changes are assessed with integrated imaging and biochemical assays. The modularity of the Hamilton deck makes the current HCS platform ideal for more sensitive organoid lines which would require a longer duration of culture or more complex preparation steps [33]. With minimal additional programming and Hamilton deck optimization, the current system is capable of standardizing and automating at least the time and labor-intensive steps for 96-well or 384-well plate-based organoid line generation which may take one month or more for brain organoid cultures.

This integration of both imaging and biochemical modalities for assessing viability can also be expanded to assess several different phenotypes in parallel. Specifically, utilization of various confocal laser colors and filters can assess drug uptake, as seen with doxorubicin HCl, cell death, cell viability, and nuclear staining altogether. Though these are the specific dyes that have been used in this study, different compounds can easily be adapted into the workflow. In addition, recent advances in organoid screening technology, including dye-free image analyses [34], may also be incorporated into the present system if staining is expected to complicate organoid growth, as is the case with DNA-intercalating agents.

Finally, this study has provided insight into the differences between the phenotypic changes provided by imaging and the biochemical changes provided by molecular assays. Overall comparison of these two methods revealed that imaging assays may be more sensitive than biochemical assays for detecting complex phenotypic changes in organoids and may provide insight into drug responses at earlier concentrations. While biochemical assays are efficient at well-based quantification of a specific metabolite such as we do for ATP here, imaging studies can provide information that is otherwise typically not observed. This is specifically useful when co-culturing different organoid lines within the same assay well. Fluorescent markers may be used to separate organoid lines and imaging software can be optimized to assess each culture individually. Here, organoids were segmented as whole structures and quantified during image analysis in 384-well plates. The rationale for this whole organoid segmentation strategy is that imaging in high-content screening is currently somewhat limited by imaging time and data storage bottlenecks; dissection at the cellular level requires both longer imaging times and higher resolution. However, this platform has been successfully adapted to segment individual cells in 3D organoids by imaging at higher magnification and with a larger number of Z-stacks [35]. Overall, the findings from these studies reveal the complexity in assessing responses to therapy. The remaining challenges lay in further process optimization, advances in organoid culture technology, and opening technological bottlenecks. As these challenges are addressed, the opportunity for dissecting phenotypic changes in high-content screening experiments will continue to grow. More specifically, advancing to 1536-well plates remains a goal due to the attractive cost efficiency of further miniaturizing experiments and screening larger libraries of compounds.

Thus, we have implemented a platform that enables functional multimodal analysis of viable 3D structures in a high content fashion, where live multiparametric morphometric analysis, paralleled with biochemical determinations provide a comprehensive phenotyping of complex cellular models during perturbation.

## Supplementary Material

Supplementary material

Supplementary material associated with this article can be found, in the online version, at doi:10.1016/j.slasd.2024.100182.
