# Supplementary material for: Development of an automated 3D high content cell screening platform for organoid phenotyping
Source: SLAS Discov. Author manuscript; Available in PMC 2025 Aug 26. (PMC12380041; doi:10.1016/j.slasd.2024.100182)
Supplement: Supplementary material [file NIHMS2106312-supplement-Supplementary_material.pdf]

Supplementary Material: Development of an automated 3D high content cell screening platform for organoid phenotyping

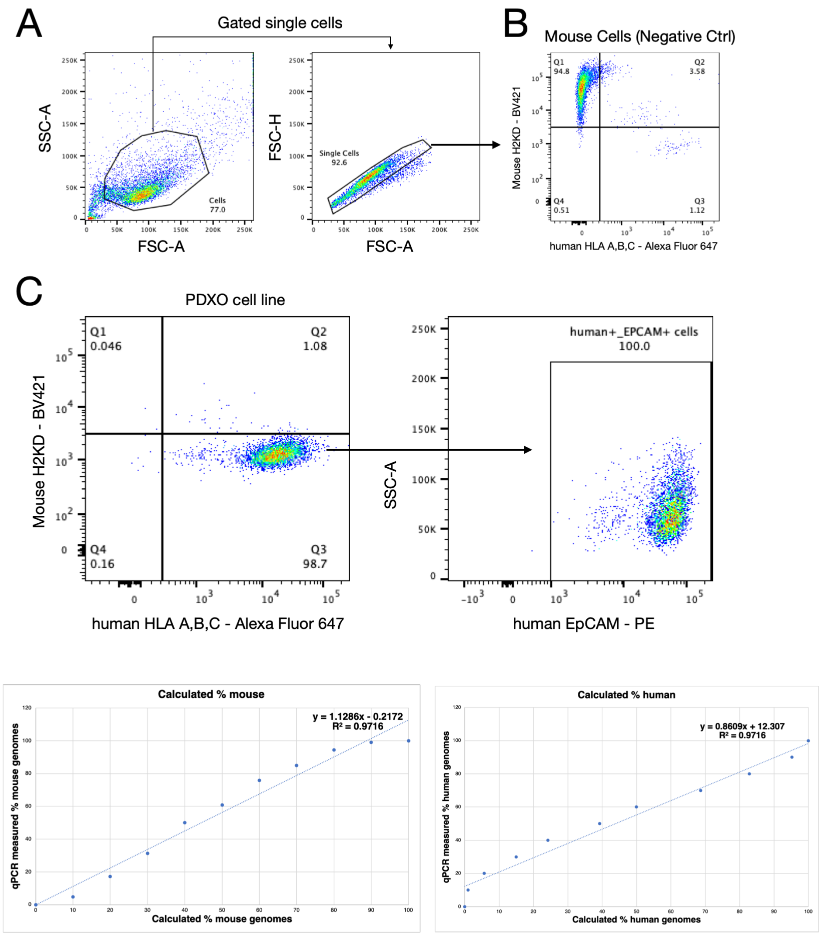

E

	%mouse	%human
**CO-1 n=1**	0.0	100.0
**CO-1 n=2**	0.0	100.0
**No template ctrl**	0.0	0.0

Supplementary Figure 1. Quality control measures ensure human and epithelial composition of PDX-derived organoids (PDXOs). **(A)** Cells are gated for single cells, **(B)** H2KDBV421 marker is used to label mouse cell as shown in control mouse cells, and in **(C)** organoid-derived cell suspension. Human HLA A,B,C, marker is used to confirm human species. **(C)** An example fluorescence-activated cell sorting plot shows that PDXO-derived cells are positive for HLA A,B,C and negative for mouse H2KD-BV421. Downstream analysis of human cells confirmed than they are of epithelial nature and express EpCAM. **(D)** Samples with defined percentages of human (HEK293 cells) and mouse DNA (mouse lung) are used to produce standard curves and quantify the **(E)** mouse and human content of CO-1 as an additional measure of quality control.

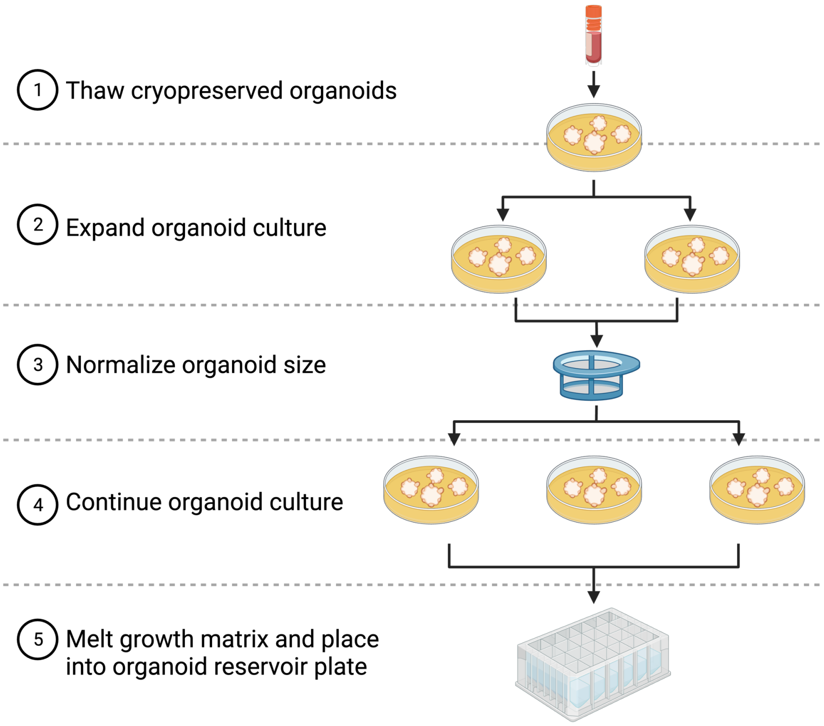

Supplementary Figure 2. Preparation of organoids for plating on the Hamilton liquid handler. **(1)** Organoids are thawed from cryopreserved stocks or derived from tissue, **(2)** expanded and **(3)** normalized by sieving through a 70 μm filter. They are then **(4)** grown for a brief period before they are **(5)** plated onto a reservoir plate for dispensing on the automated system.

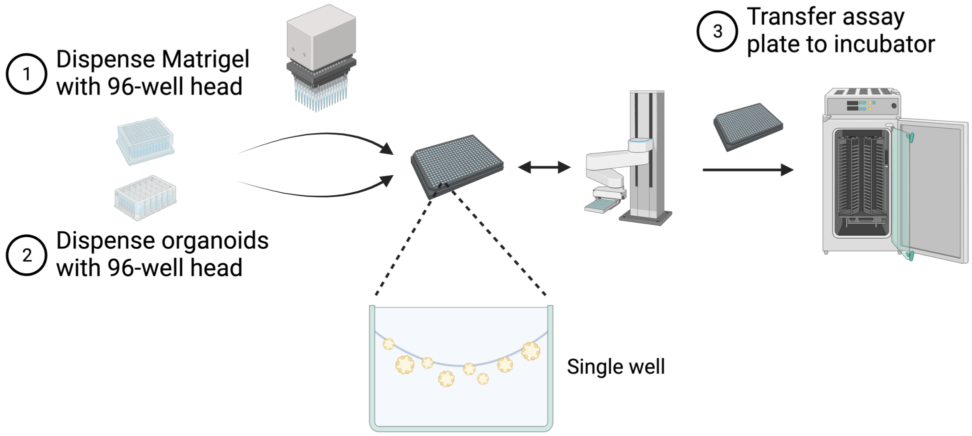

Supplementary Figure 3. Matrigel and organoid plating is coordinated to allow robotic transfer into an automation-compatible incubator. **(1)** Matrigel is dispensed with a 96-well head and incubated on a thermal plate to allow solidification followed by **(2)** dispensing of organoids. **(3)** The Assay plate is then transferred by a robotic arm into an automation-compatible incubator.

	Aspirate	Dispense
**Material:** Matrigel, **Tip:** 300 µL, **Dispense mode:** Surface part volume		
**Flow rate**	50 ul/s	10 ul/s
**Mix flow rate**	10 ul/s	1
**Air transport volume**	0 ul	0 ul
**Blowout volume**	0 ul	0 ul
**Settling Time**	2 secs	1 sec
**Material:** BSA rinse, **Tip:** 300 µL, **Dispense mode:** Jet empty tip		
**Flow rate**	100 ul/s	45 ul/s
**Mix flow rate**	90 ul/s	
**Air transport volume**	0 ul	5 ul
**Blowout volume**	10 ul	0 ul
**Material:** Water, **Tip:** 300 µL, **Dispense mode:** Jet empty tip		
**Flow rate**	100 ul/s	180 ul/s
**Mix flow rate**	100 ul/s	
**Air transport volume**	5 ul	5 ul
**Blowout volume**	30 ul	30 ul
**Material:** High volume water, **Tip:** 1000 µL, **Dispense mode:** Jet empty tip		
**Flow rate**	250 ul/s	400 ul/s
**Mix flow rate**	250 ul/s	
**Air transport volume**	5 ul	5 ul
**Blowout volume**	40 ul	40 ul
**Material:** Drug stocks, **Tip:** 50 µL, **Dispense mode:** Surface empty tip		
**Flow rate**	100 ul/s	120 ul/s
**Mix flow rate**	75 ul/s	75 ul/s
**Air transport volume**	0 ul	0 ul
**Blowout volume**	1 ul	1 ul
**Material:** Drug dilutions, **Tip:** 1000 µL, **Dispense mode:** Surface empty tip		
**Flow rate**	250 ul/s	120 ul/s
**Mix flow rate**	120 ul/s	120 ul/s
**Air transport volume**	5 ul	5 ul
**Blowout volume**	0 ul	0 ul

	Aspirate	Dispense
**Material:** Randomization, **Tip:** 1000 µL, **Dispense mode:** Surface empty tip		
**Flow rate**	100 ul/s	120 ul/s
**Mix flow rate**	100 ul/s	100 ul/s
**Air transport volume**	5 ul	5 ul
**Blowout volume**	0 ul	0 ul
**Material:** Drugs to reservoir plate **Tip:** 50 µL, **Dispense mode:** Jet empty tip		
**Flow rate**	100 ul/s	60 ul/s
**Mix flow rate**	100 ul/s	1 ul/s
**Air transport volume**	5 ul	5 ul
**Blowout volume**	10 ul	5 ul
**Material:** Drugs to assay, **Tip:** 50 µL, **Dispense mode:** Surface empty tip		
**Flow rate**	100 ul/s	75 ul/s
**Mix flow rate**	75 ul/s	75 ul/s
**Air transport volume**	1 ul	1 ul
**Blowout volume**	10 ul	8 ul
**Material:** Dye dispensing, **Tip:** 10 µL, **Dispense mode:** Surface empty tip		
**Flow rate**	25 ul/s	35 ul/s
**Mix flow rate**	25 ul/s	35 ul/s
**Air transport volume**	0 ul	0 ul
**Blowout volume**	10 ul	10 ul
**Settling Time**	2 secs	1 sec
**Material:** CellTiter-Glo, **Tip:** 300 µL, **Dispense mode:** Surface empty tip		
**Flow rate**	80 ul/s	100 ul/s
**Mix flow rate**	80 ul/s	80 ul/s
**Air transport volume**	5 ul	5 ul
**Blowout volume**	10 ul	8 ul

Supplementary Figure 4. Liquid class definitions outlining aspiration and dispensing parameters for different materials and protocol steps

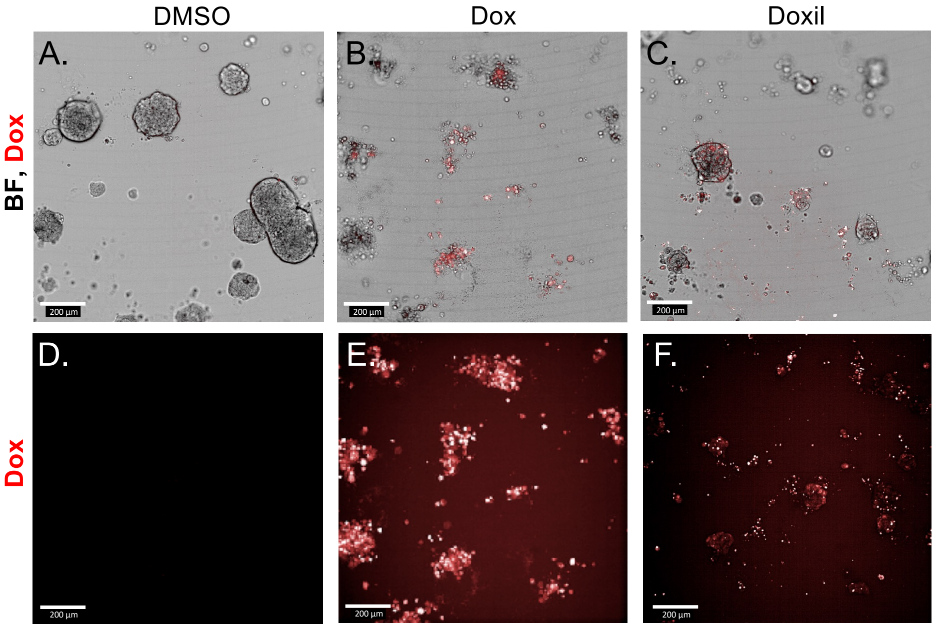

Supplementary Figure 5. Confocal imaging with the Opera Phenix High-Content Imaging System shows localization of doxorubicin (red) in bladder tumor organoids. **(A, B, and C)** show brightfield images of bladder tumor organoids with overlapped doxorubicin (red) imaging. **(D, E, and F)** show the same cultures with doxorubicin (red) imaging only. **(A)** and **(D)** show 0.5% DMSO treated organoids. **(B)** and **(E)** show doxorubicin treated organoids. **(C)** and **(F)** show Doxil treated organoids. Cultures are imaged after 3 days. Cultures treated with doxorubicin are at a concentration of 20 µM. Scale bars = 200 µm.

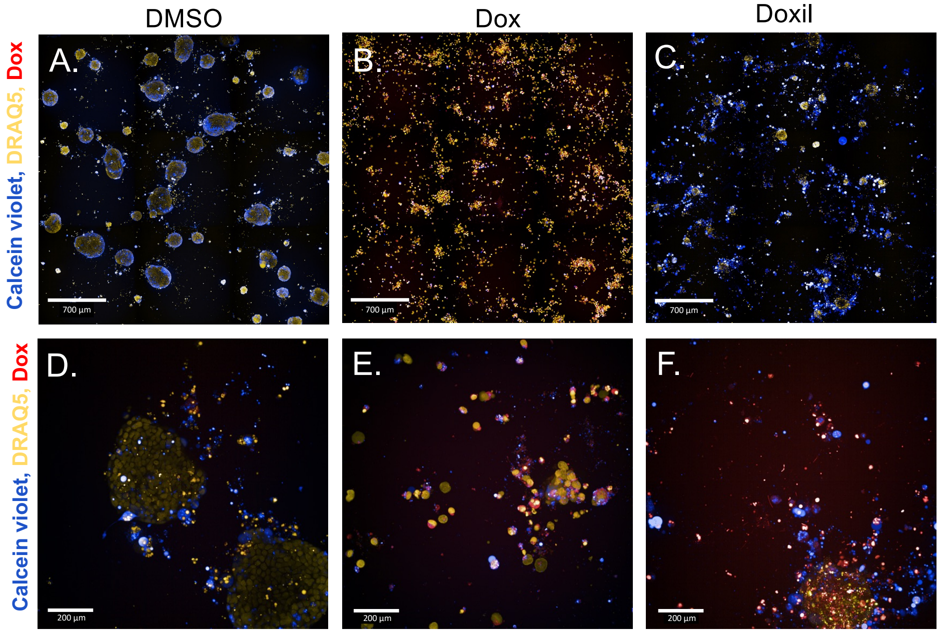

Supplementary Figure 6. Confocal imaging with multiple channels allows live-dead imaging of bladder tumor organoids following # days of incubation. Calcein violet (blue) is a cell permeant dye used to label live cells, DRAQ5 (yellow) is a cell permeant dye that stains DNA. **(A, B, C)** at 4X show bladder tumor organoids stained with calcein violet and DRAQ-5 in wells treated with 0.5% DMSO, Dox, or Doxil, respectively with scale bars = 700 µm. Doxorubicin (red) is tracked as well. **(D, E, F)** at 10X show single-field images of the same culture conditions with scale bar = 200 µm. Cultures treated with doxorubicin are at a concentration of 20 µM.

Sample ID	Z-Average(d.nm)	PDI	Drug Morphology
Doxil	83.8 ± 0.21	0.032 ± 0.005	Linear, Elongated
Formulation 1	85.8 ± 1.26	0.067 ± 0.006	Linear

Supplementary Figure 7. Particle characteristics of Doxorubicin-loaded (2 mg/mL) liposomes

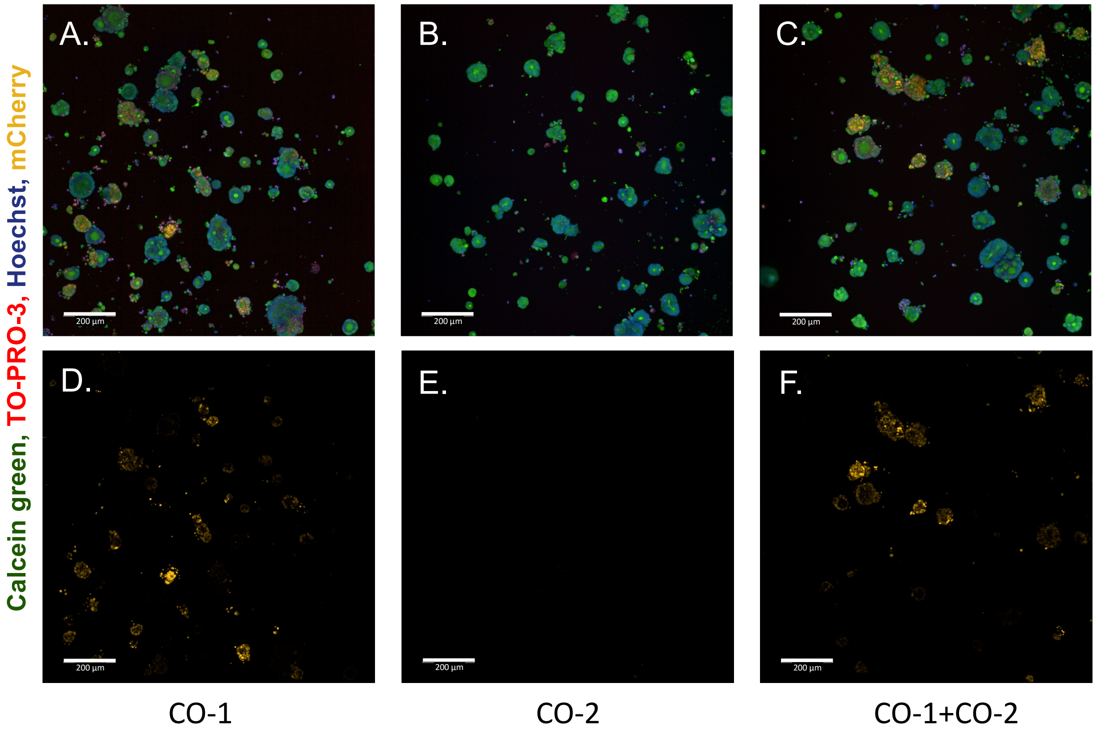

Supplementary Figure 8. Imaging of colorectal organoids 24 hr after plating on Matrigel. Calcein green is used to stain live cells while TO-PRO-3 (red) is used to stain dead cells. Yellow denotes mCherry positive organoids and the blue stain is Hoechst, a fluorescent dye that stains DNA. **(A)** is a culture of mCherry (+) CO-1 organoids. **(B)** is a culture of CO-2 organoids, and **(C)** is a co-culture of CO-1 and CO-2 organoids in equivalent amounts. **(D, E, and F)** are images of the mCherry (+) organoids in each culture. The mCherry (+) organoids in panel **(F)** are CO-1 organoids. These images were gathered on Day 1 of the assay, ~24hr after initial plating of the organoids, at 10X magnification of 1 field of view. Scale bar = 200 μm.
